# MULTIMORBIDITY PATTERNS AND COGNITIVE CHANGE AMONG MEXICAN OLDER ADULTS IN THE MEXICAN HEALTH AND AGING STUDY

**DOI:** 10.1093/geroni/igad104.3481

**Published:** 2023-12-21

**Authors:** Nicholas Bishop, Anda Botoseneanu, Sheila Markwardt, Priscilla Zambrano, Kealie Walker, Ana Quiñones

**Affiliations:** University of Arizona, Tucson, Arizona, United States; University of Michigan, Ann Arbor, Michigan, United States; Oregon Health & Science University, Portland, Oregon, United States; University of Arizona, Tucson, Arizona, United States; University of Arizona, Tucson, Arizona, United States; Oregon Health & Science University, Portland, Oregon, United States

## Abstract

Multimorbidity (≥ 2 chronic conditions) increases the risk of non-normative cognitive decline. Among Mexican older adults, little is known about 1) the prevalence of multimorbidity, 2) patterns of multimorbidity, and 3) the relationship between multimorbidity patterns and cognitive decline. We used representative data from the Mexican Health and Aging Study to estimate prevalence of multimorbidity, identify common multimorbidity patterns, and examine associations between multimorbidity patterns and cognitive change from 2012–2015 in Mexican adults aged ≥ 65 (n=5,079; mean age: 72.2 (SD=6.1)). Chronic conditions including hypertension, diabetes, cancer, lung disease, heart attack, stroke, and arthritis were measured in 2012, and cognitive function was assessed in 2012 and 2015 using the Cross Cultural Cognitive Examination (CCCE; range:3–96). We used descriptive analyses to estimate multimorbidity prevalence and identify disease patterns, then used weighted linear regression (adjusted for age, sex, martial/partnership status, education, literacy, and household income and wealth) to estimate associations between multimorbidity patterns and cognitive change (modeled as CCCE-2015 regressed on CCCE-2012). In 2012, 50.7% of respondents reported multimorbidity and average CCCE score was 46.6 (SD=17.2). From 2012–2015, CCCE decreased on average 2.7 units (SD=13.1). Disease patterns with ≥ 2% prevalence included hypertension+depression (6.0%), hypertension+arthritis (5.9%), hypertension+diabetes (5.8%), hypertension+arthritis+depression (5.4%), hypertension+diabetes+depression (2.8%), hypertension+diabetes+arthritis (2.7%), and arthritis+depression (2.1%). Compared to those reporting no conditions, respondents reporting arthritis+depression (b=-3.88, CI=-6.11:-1.66), hypertension+diabetes (b=-3.72, CI=-5.33:-2.11), or hypertension+diabetes+depression (b=-3.41, CI=-5.32:-1.21) experienced greater cognitive decline. Our results suggest diabetes and depression are common elements of multimorbidity associated with cognitive decline in Mexican older adults.

